# Motion After‐Effects Induced by Dynamic Illumination in Crab Vision

**DOI:** 10.1002/ece3.71426

**Published:** 2025-05-10

**Authors:** Christian Drerup, James E. Herbert‐Read, Martin J. How

**Affiliations:** ^1^ Department of Zoology University of Cambridge Cambridge UK; ^2^ Department of Biosciences Durham University Durham UK; ^3^ School of Biological Sciences University of Bristol Bristol UK

**Keywords:** background motion, *Carcinus maenas*, caustics, sensory ecology

## Abstract

Motion detection is an elementary aspect of most animal visual systems. However, many environments are prone to background motion, which might disrupt the ability of visual systems to detect relevant motion cues. While in humans, background motion can disrupt the detection of visual cues even after the moving background component has ceased, it remains unknown whether natural forms of background motion might also affect other animal visual systems. Here, we test whether prior exposure to naturally occurring ‘caustics’, a form of dynamically moving light patterns commonly found in shallow aquatic environments, can have a persisting effect on an animal's motion detection abilities even after the caustic exposure has stopped. To do this, we established the response probability of the shore crab *Carcinus maenas* to computer‐generated expanding disc stimuli mimicking an approaching predator after exposure to either static or moving caustic scenes. Prior exposure to moving caustics had a short‐term persisting effect on visual perception in *C. maenas* , reducing crabs' likelihood to respond to an approaching predator for at least 2 s after the moving caustics had ceased. Our study shows that even after an exposure period to background motion has ended, the visual response rates in *C. maenas* can still be reduced for a short period owing to the prior exposure. While this so‐called ‘historical effect’ may derive from an adaptation of the crab's visual system to the caustic background motion, we discuss whether it may have survival consequences for this crustacean species.

## Graphical abstract

While in humans, background motion can disrupt the detection of visual cues even after the moving background component has ceased, it remains unknown whether natural forms of background motion might also affect other animal visual systems. Here, we test whether prior exposure to naturally occurring ‘caustics’, a form of dynamically moving light patterns commonly found in shallow aquatic environments, can have a persisting effect on the motion detection abilities of the shore crab *Carcinus maenas* even after the caustic exposure has stopped. Prior exposure to moving caustics had a short‐term persisting effect on visual perception in crabs, reducing their likelihood to respond to an approaching predator for at least 2 s after the moving caustics had ceased.

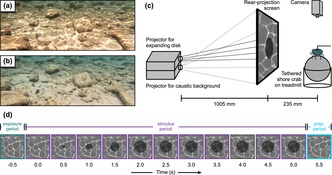

## 1 Introduction

Many animals rely on their visual systems to detect information about their predators, prey or conspecifics. Although animal eyes can vary in both anatomy and complexity, some functions are common across visual systems, highlighting their ecological importance for an animal's survival and reproductive success (Land and Nilsson 2012). One of these functions is the ability to detect motion, which is used for a wide range of behavioural processes such as navigation, postural stability, prey capture or predator detection (Eckert and Zeil 2001; Srinivasan et al. 1999). Many natural environments, however, are prone to background motion, for example through wind‐blown vegetation or the movements of clouds or aquatic surfaces, such as rivers or waterfalls, creating dynamic visual elements across sometimes large parts of the visual scene. Such background motion can act as a source of visual noise, impairing the ability of animals to detect and respond to motion cues (Attwell et al. 2021; Matchette et al. 2020; Venables et al. 2022). In particular, background motion can interfere with animal perception through two processes, either by masking other moving stimuli within the scene (Matchette et al. 2018, 2019) or by distracting an animal, thereby limiting its ability to detect, respond, or process visual information (Dominoni et al. 2020).

Although background motion can interfere with many behavioural and perceptual processes in animals, previous studies have not unravelled whether these impacts derive exclusively from the interference that background motion can create at the moment of cue detection (irrespective of any prior exposure to the moving background scene) or whether prior exposure to background motion also affects animal visual processing. For example, visual systems can perform neural adaptations to continuous background motion, thereby increasing an animal's likelihood of detecting other moving stimuli (Webster 2015). In particular, owing to adaptation, individuals that experience prolonged exposure to a moving background might be more likely to detect and respond to a moving cue (e.g., a moving predator) against a moving background than individuals that have had less time to adapt to that background. However, prior exposure to background motion can also affect animal visual systems after the background motion has stopped. In humans, for example, shifting the gaze from a scene with continuous exposure to a moving stimulus (e.g., a waterfall) to a stationary background can create the illusion of stationary objects moving in the direction opposite to the previous moving stimulus (Anstis et al. 1998), a phenomenon known as the ‘waterfall illusion’ (Addams 1834). These ‘historical effects’ of background motion can impair the detection of information, thereby potentially interfering with perceptual and decision‐making processes (Gallagher et al. 2021). Looking beyond human perception, such historical effects of background motion have also been established in primates; however, these studies exposed animals to non‐natural moving stimuli, such as drifting sine wave gratings (Glasser et al. 2011; Kohn and Movshon 2003). Whether naturally occurring and ecologically relevant background motion, such as the moving light patterns deriving from dynamic lighting, can induce a historical effect on animal visual systems remains unknown.

**FIGURE 1:**
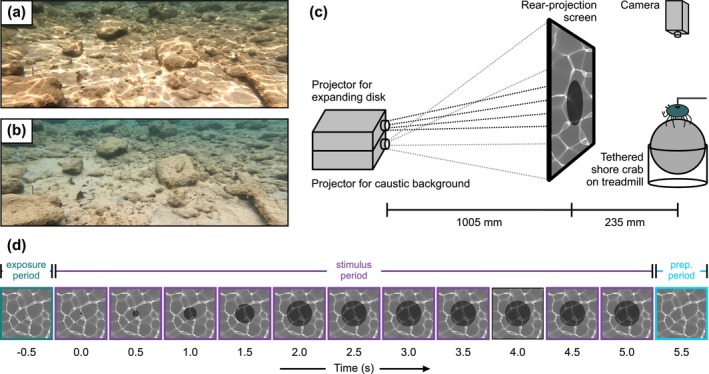
Example of caustic exposure and experimental set‐up. (a, b) Dynamic lighting in the form of caustics, with (a) shallow marine habitat exposed to caustic lighting and (b) the same habitat after caustics have ceased (approximately 4 s later). (c) Schematic drawing of the experimental set‐up, with tethered shore crabs walking on a treadmill while facing a rear‐projection screen, which shows expanding disc presentations on a caustic background. (d) Visualisation for expanding disc presentation during the stimulus period in Experiment 1. During the exposure period, crabs were only shown a moving or static caustic background, whereas immediately with the beginning of the stimulus period, crabs were exposed to a caustic background as well as an approaching predator‐like expanding disc stimulus. Here, the disc size expanded over a 2‐s‐long period following a geometric expansion profile (see Section 2.2, Experimental Set‐Up, for detailed description) and remained visible at full expansion for another 3 s, resulting in a 5‐s‐long stimulus period. After the stimulus period, the preparation period (prep. period) began, which included exposure to the caustic condition of the stimulus period for approximately 20 s to allow preparation of the following treatment (see Section 2.6, Experimental Protocol).

One common form of background motion in shallow aquatic habitats is ‘caustics’ (also termed caustic flicker; Figure 1a,b). Consisting of mesh‐like patterns of high‐intensity light bands moving across the substrate, caustics result from the fluctuation of focal points of light beams when refracted by surface waves (Lock and Andrews 1992). In environments prone to caustic lighting, animals might use the apparent motion cues provided by this form of dynamic illumination to mask their own movements, which in turn can increase the difficulty for a predator or prey to detect them (Matchette et al. 2018). Indeed, exposure to caustics can disrupt the prey or predator detection of Picasso triggerfish ( *Rhinecanthus aculeatus* ; Matchette et al. 2020), three‐spined sticklebacks ( *Gasterosteus aculeatus* ; Attwell et al. 2021), shore crabs ( *Carcinus maenas* ; Venables et al. 2022) and European cuttlefish ( *Sepia officinalis* ; Venables et al. 2022). Although dynamically moving caustic light patterns can impair the visual systems of aquatic animals (Attwell et al. 2021; Matchette et al. 2020, but see Drerup et al. 2023; Drerup, How, et al. 2024; Venables et al. 2022), it remains unknown whether solely the background motion at the moment of cue presentation (irrespective of any prior exposure to caustics) reduces the ability to respond to these objects in these species, or whether additional prior exposure to caustics might either improve or further impair the visual perception of these animals. Indeed, caustics can appear and disappear in rapid succession due to cloud coverage or fluctuating wind conditions, exposing animals in shallow marine environments to unpredictable phases of background motion. Caustics, therefore, represent a naturally occurring form of background motion that has the potential to elicit a historical effect on the visual systems of marine animals.

In this study, we investigated the temporal effects of background motion on the ability of shore crabs ( *C. maenas* ) to detect predators. This brachyuran crab species is commonly found in coastal environments such as intertidal and shallow subtidal zones (Crothers 1968) where it relies on its visual system, consisting of a pair of compound eyes, for a multitude of behavioural tasks, including detecting predators, catching prey and finding conspecifics (Cronin and Feller 2014). As such, their eyes are tuned to detect different forms of motion in the visual field (Horseman et al. 2011). Considering that their natural habitats are prone to rapidly appearing and disappearing caustic lighting patterns that can impair this species' visual perception (Venables et al. 2022), *C. maenas* offers a suitable model to establish the temporal effects of background motion induced by dynamic lighting on the ability to detect moving stimuli. By exposing individuals of *C. maenas* to changing visual scenes consisting of computer‐generated simulations of either moving or static caustic patterns while simultaneously presenting them with looming stimuli mimicking an approaching predator, we assessed whether exposure to moving caustics could elicit an ecologically relevant historic effect on motion detection in crabs.

## 2 Material and Methods

### 2.1 Study Organism

Shore crabs *C. maenas* were collected from Clevedon Beach (UK; 51°26′18.0″ N 2°51′56.7″ W) and kept in individual compartments (180 × 130 × 60 mm) in a circulating shallow aquarium filled with artificial sea water (Tropic Marin AG, Wartenberg, Germany) at a salinity of 35 ppt. All holding compartments were exposed to light cycles matching the natural photoperiod of the experimental period (May–June). Shore crabs were fed twice a week with defrosted mussels or prawns. All individuals were returned to the collection site within a week of collection.

As we used wild‐caught individuals of *C. maenas* , we were unable to directly control for individual differences in previous experience to caustic lighting exposure. However, our study did not aim to assess the response of *C. maenas* to novel visual stimuli but rather to establish how the visual systems of *C. maenas* cope with naturally occurring background motion in its habitat, which eliminates the need to test naïve individuals. With this in mind, we aimed to keep the effect of individual experience as minimal as possible by only collecting individuals of comparable sizes from the same geographic location (200 m wide beach segment) within a short time frame (3 weeks), while further controlling for individual variation through including individual ID as a random effect in our statistical models (see Section 2.8, Statistical Analysis).

### 2.2 Experimental Set‐Up

Crabs were tethered on top of a spherical treadmill consisting of a Styrofoam ball (diameter = 120 mm) suspended on a cushion of air supplied from a compressed air tap (Figure 1c). Tethering involved gluing a piece of Velcro onto the dorsal side of the crab carapace using cyanoacrylate glue and a complementary piece of Velcro to a metal rod horizontally mounted above the treadmill. This allowed the crabs to walk freely but restricted their translational or rotational movement. A video camcorder (HC‐X900, Panasonic Corporation, Osaka, Japan) was mounted 375 mm above the treadmill to record the behaviour of the crabs throughout each trial.

Crabs faced a custom‐made stimulus screen at a distance of 235 mm that allowed a simultaneous yet independent display of caustic backgrounds and predator‐like approaching stimuli. This screen consisted of two digital projectors (PA503S, ViewSonic Corporation, Brea, USA) stacked on top of each other, with their images overlaying onto a semi‐transparent 300 × 300 mm rear‐projection screen (0.5 diffusor, Lee Filters, Andover, UK) at a distance of 1005 mm (Figure 1c). One projector, connected to a laptop (Ideapad 310, Lenovo Group Limited, Hong Kong, VR China), displayed a computer‐generated animation of a caustic pattern that was rendered using Caustics Generator Pro (Dual Heights; www.dualheights.se/caustics/; detailed software settings are presented in Table A1). The caustic pattern consisted of 200 unique frames continuously looped at a frame rate of 30 frames/s, resulting in a single loop duration of 6.66 s. The second projector, connected to a second laptop (G3, Dell Technologies Inc., Round Rock, USA), was used to project an expanding black disc stimulus generated using a bespoke MATLAB script (R2021a, MathWorks, Natick, USA). Expanding black disc stimuli are a common experimental approach to mimic the looming appearance of an approaching predator (Calanni et al. 2024; Schiff et al. 1962), which elicits a strong innate freezing response in *C. maenas* (Drerup and How 2021; Venables et al. 2022; see 2.7, Response scoring). The black disc expanded over a 2‐s‐long period from a visual angle of 0° to ~33° (0–139 mm in diameter), following a geometric expansion profile (i.e., the angular rate of expansion of the disc matched that induced by a physical object approaching at a constant speed), and remained visible at full expansion for another 3 s (Figure 1d). As ‘broad‐fronted’ crabs largely lack acute zones in their compound eyes (Zeil et al. 1986), our set‐up exposed tethered *C. maenas* individuals with expanding discs stimuli in their eye equator, with such stimuli in this part of the field of view having been shown to elicit strong anti‐predatory responses (e.g., Drerup and How 2021; Venables et al. 2022). Both projectors were run at refresh rates of 60 Hz, which lies above the critical flicker fusion frequency of crabs (approximately 30–50 Hz; Grober 1990; Layne et al. 1997). Audio beeps produced by the MATLAB script at the beginning and end of each expanding disc presentation were fed into the audio stream of the video camcorder to synchronise crab's response to the stimulus. To minimise external disturbances, a white cubicle consisting of a 40 × 40 × 40 cm wide photography tent was placed around the crab treadmill, with two openings allowing crabs to face the rear‐projection screen, as well as the camera to film the crabs' behaviour.

Using our set‐up (comprising two projectors and overlaying their images onto a rear‐projection screen) had three advantages. First, using two independent animation streams to display the caustics through one projector and the expanding disc stimulus through the other projector enabled us to start/stop either animation independently of each other. This allowed us to time stimulus presentations to phases in which shore crabs were moving, which was pertinent to our response scoring approach (see 2.7, Response Scoring). Second, the use of digital light processing (DLP) projectors in our set‐up allowed us to display both the caustic backgrounds as well as the expanding disc stimuli as intensity‐based cues only, without any unwanted fluctuations or artefacts in the polarisation of light. *C. maenas* is capable of mitigating the impacts of caustic lighting on its ability to detect objects if the latter are polarised (Venables et al. 2022), and so our experimental set‐up ensured that any behavioural responses observed in this study are solely elicited by the impact caustics might have on this species' intensity‐based visual channels. Third, by projecting both the caustic animation and the expanding disc stimulus onto the rear‐projection screen, both presentations appeared to the crabs on the same visual plane. This approach allowed us to superimpose the caustics onto both the background as well as the expanding disc stimulus, thereby approximating how natural caustics would interact with an approaching object (e.g., a predator) against a background. Our experimental design, therefore, represents a scenario in which a crab is approached by a predator viewed against a vertical or sloped background, such as may be found in shallow coastal habitats or large rock pools. Although our set‐up thus generates motion in the form of dynamically moving caustic light patterns across both the visual background and foreground, for simplicity we refer to our caustic treatments as background motion throughout the remainder of the manuscript.

### 2.3 Contrast Measurements

**FIGURE A1:**
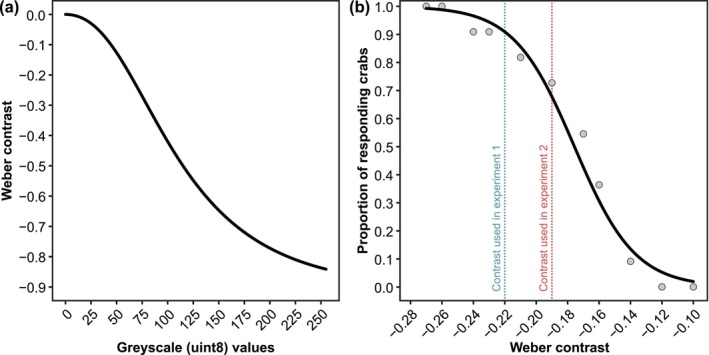
Contrast measurements. Our experimental set‐up (Figure 1c) consisted of two projectors stacked on top of each other, with their images overlaying onto a semi‐transparent rear‐projection screen. By using one projector to cast a static caustic image and the other projector to cast backgrounds varying in their greyscale (uint8) values onto the rear‐projection screen, (a) shows the Weber contrast between a greyscale value of 0 (used for the stimulus disc) and all 256 greyscale images (used for the stimulus background). (b) Pilot experiment testing the response probability of *Carcinus maenas* (*n* = 11) for 11 expanding disc stimuli varying in their Weber contrast against a static caustic image. The responses of the shore crabs to the looming stimuli were established using a binary scoring system (see Section 2.7, Response Scoring). From these data, for Experiment 1 we deemed a Weber contrast of −0.22 (green line) to be the lowest contrast that elicited a robust (~90%) response rate to an expanding disc against a static caustic background but correspondingly a lower response rate against moving caustics. To fall nearer to the crab's response threshold, we opted for a Weber contrast of −0.19 (red line) for Experiment 2.

Our treatments consisted of a caustic animation overlaid by an approaching predator‐like stimulus consisting of a black disc (uint8 value = 0) expanding on a background with the same or a lighter greyscale value (uint8 value between 0 and 255). To calculate the contrast between the predator‐like stimulus (expanding disc overlaid by caustics) and the background (greyscale background overlaid by caustics), we first projected both a still frame of the caustic animation (using the first projector) as well as a series of 256 frames consisting of all 256 uint8 greyscale values (using the second projector) onto the rear‐projection screen. Using a spectrometer (HDX) coupled to a 400 μm bare optic fibre with a cosine corrector (R400‐7‐UV‐VIS; all Ocean Insight, Orlando, USA), we then measured the radiance of a subset of the overlaid image, covering approximately half of the screen area, for all combinations of caustic background and each of the 256 greyscale values. Radiance measurements were capped to the wavelength range of 400–700 nm, which covers the spectral sensitivity of *C. maenas* (Bruno et al. 1973; Wald 1968). We then used these radiance measurements to calculate the Weber contrast of all 256 greyscale values (Figure A1a), used for the background of the stimulus projection, against the darkest greyscale image (uint8 value = 0), used for the expanding discs of the stimulus projection, using the following formula:

$$\frac{{\text{Radiance}}_{\text{disk}}-{\text{Radiance}}_{\text{background}}}{{\text{Radiance}}_{\text{background}}}$$

To determine an appropriate experimental range of stimulus‐background contrasts, we conducted a pilot experiment testing the response rate of 11 *C. maenas* individuals (not reused in Experiment 1 or 2) to 11 expanding discs with Weber contrasts between −0.10 and −0.27 against a static caustic background, presented in a randomised block design to avoid an order bias. The responses of the shore crabs to the looming stimuli were established using a binary scoring system (see Section 2.7, Response Scoring) and are displayed in Figure A1b.

### 2.4 Experiment 1

To establish the temporal effects of background motion on the response of *C. maenas* , we exposed 56 individuals (carapace width: 32 ± 9 mm [mean ± 1 SD]) each to eight different treatments. Each treatment started with a ≥ 90 s long ‘exposure period’ to either a static or moving caustic background and was immediately followed by the ‘stimulus period’, which could either be a continuation of the same caustic condition or an abrupt change into the other caustic condition. Within the stimulus period, crabs were then exposed to an expanding disc stimulus, which started simultaneously with the change from the exposure to the stimulus period, thereby reaching its full size 2 s into the stimulus period (Figure 1d). In four of the eight treatments of this experiment, the expanding discs were projected at a Weber contrast of −0.22 (Figure A1b), which was deemed to be the lowest contrast that elicited a robust (~90%) response rate to an expanding disc against a static caustic background but correspondingly a lower response rate against moving caustics (following Venables et al. 2022). In the other four treatments, the expanding discs were presented at a Weber contrast of 0.00, which resulted in an ‘invisible’ expanding disc and was used as a control to establish whether any observed behavioural responses derived indeed from exposure to the approaching predator‐like stimuli and not from potential changes in the visual background, thereby assessing the proportion of false positive responses. We opted for presenting the crabs an invisible disc instead of no disc at all to ensure that any observed responses in crab behaviour are not elicited by deviations in our experimental protocol (e.g., the manual starting of an expanding disc display by the experimenter or any disruptions in the caustic animations caused by the presentation of an expanding disc). In total, there were eight treatments, with variations of static or moving caustics in both the exposure and stimulus periods, as well as the contrast (‘visible’ or ‘invisible’) of the expanding disc. We gave each treatment a three‐letter code, with the first and second letter stating the caustic condition in the exposure and then stimulus period (‘M’ for moving caustics, ‘S’ for static caustics), and the third letter stating the visibility of the expanding disc (‘V’ for a visible disc with a Weber contrast of −0.22, ‘I’ for an invisible disc with a Weber contrast of 0.00). Each crab (*n* = 56) received all eight treatments in one trial and was only tested once. To ensure that the order of the treatments presented to an individual within a trial did not affect its response likelihood, we created 56 unique orders of the eight treatments in a balanced randomised block design, with each crab thus receiving a unique order of the eight treatments within its trial.

### 2.5 Experiment 2

Following the results of Experiment 1, we determined for how long prior exposure to moving caustics reduced the response rate for looming stimuli after the caustics had stopped. To do so, we exposed 48 new crabs (i.e., not used in Experiment 1; carapace width: 31 ± 8 mm [mean ± 1 SD]) to eight different treatments. Each treatment started with a ≥ 90 s long ‘exposure period’ to either a static or moving caustic background and was immediately followed by the ‘stimulus period’, which in this experiment always consisted of a static caustic background. In the stimulus period, we then presented expanding discs with different starting points, namely after 0 s (thus starting with the change from exposure to stimulus period) or with an offset of 1, 2, 3, 4 or 5 s. Based on information from Experiment 1, we reduced the Weber contrast of the visible expanding discs in Experiment 2 from −0.22 of −0.19 to fall nearer to the crab's response threshold (Figure A1b), while invisible discs were still presented at a Weber contrast of 0.00. Each of the eight treatments used in this experiment were assigned an alphanumerical code, following the same system as the previous experiment, but with the addition of a digit referring to the offset (in seconds) at which the disc started to expand (in relation to the start of the stimulus period). Each crab (*n* = 48) was tested once in this experiment and received all eight treatments in one trial. We created 48 unique orders of the eight treatments in a balanced randomised block design, thereby ensuring that each crab would receive a unique order of the eight treatments within its trial.

### 2.6 Experimental Protocol

Each trial started by tethering a crab on the treadmill and leaving it for 180 s to acclimate to a grey static background with the same brightness as the caustic backgrounds. After this period, we changed the background to the first caustic condition of the exposure period of the first treatment. Each treatment started with an at least 90‐s‐long exposure period, followed by the stimulus period. Throughout all trials of both Experiment 1 and Experiment 2, the static caustic background images presented to the crabs were randomly chosen from the 200 frames of our caustic animation, thereby ensuring variability and preventing any artefacts that could have derived from choosing one static image across all trials. As our response scoring relied on animals walking when exposed to the expanding discs, we extended the duration of the exposure period for trials in which a crab was stationary until the crab started to walk for at least 5 s, which was usually the case within < 10 s. After each treatment, crabs continued to be exposed to the caustic condition of the stimulus period for a short duration (~20 s; ‘preparation period’), thereby allowing us to prepare the settings for the subsequent treatment in our MATLAB script before starting the next treatment. In total, each treatment took approximately 2 min to complete, resulting in a total trial length of less than 20 min.

### 2.7 Response Scoring

All video recordings were imported into MATLAB. Here, we used a bespoke script to identify the audio beeps fed into the audio stream of each video at the beginning and end of each presented expanding disc. Using these audio markers, we cropped the video recording of each crab into eight individual video clips, one for each treatment within a trial. Each of these video clips had a duration of 15 s, capturing the crab behaviour from 5 s before until 5 s after the expanding disc display (5 s). In the bottom‐right corner of each of these videos, we added a temporally aligned animation of the expanding disc display, with the animations for both the visible and invisible expanding disc treatments being identical. These individual video clips thus solely showed the crab behaviour during an expanding disc presentation but excluded any indication about the caustic condition in the exposure or stimulus period or the contrast/visibility of the expanding discs, thereby allowing us to score the response behaviour of *C. maenas* blind.

Moving individuals of *C. maenas* display a robust response to low‐contrast expanding disc stimuli by drastically slowing down or stopping their movement (Drerup and How 2021; Venables et al. 2022). Our study focussed on whether crabs respond (i.e., the absolute responses) to an approaching predator‐like stimulus based on previous and current caustic exposure. Therefore, the responses of *C. maenas* to the expanding discs in our experiments were scored as binary response data, with crabs either responding to the expanding disc (1; ‘freezing’, indicated by stopping (or drastically slowing down) their movements) or not responding to the expanding disc (0; ‘walking’, indicated by continuing their walking movement). Due to using low‐contrast stimuli in our experiment, we only assessed the crabs' walking behaviour, whereas other behavioural features often seen in response to higher contrasting stimuli (e.g., claw movements; Drerup and How 2021) were not considered for our scoring approach. Behavioural responses were only included when they occurred within the expansion phase of the presented discs. All videos were scored by the same observer.

### 2.8 Statistical Analysis

All statistics were performed in R v. 4.2.2 (R Core Team 2023) and included generalised linear mixed‐effect models (GLMMs) with binomial family error structures from the package *lme4* (Bates et al. 2015). We checked the assumptions for all GLMMs using the *DHARMa* package (Hartig 2022). Significant effects of each factor within a model were determined using the ‘drop1’ call from the *lme4* package (Bates et al. 2015). In cases where treatment had a significant effect on a response measure, we used the *emmeans* package (Lenth 2022) along with custom‐written contrasts to compute the pairwise differences only between predefined treatments. Here, we corrected for multiple testing using the Benjamini–Hochberg method (Benjamini and Hochberg 1995), which controls the false discovery rate using a sequential modified Bonferroni correction. All visualisations were rendered using *ggplot2* (Wickham 2016).

For Experiment 1, we first established whether changes in the caustic conditions from the exposure to stimulus period elicited responses in the crabs even in the absence of the visible disc (i.e., false positives). We tested (1) whether the proportion of responding crabs differed between each treatment with invisible expanding discs (0.00 Weber contrast; SSI, MSI, SMI and MMI). To do so, we ran a GLMM with the response to the expanding disc as a binary response variable (0: not responded; 1: responded), treatment as a fixed effect and individual crab ID as a random effect. Subsequently, we calculated the pairwise differences between these four treatments using custom‐written contrasts. We also tested (2) whether the proportion of responding crabs differed between treatments with a visible (−0.22 Weber contrast; SSV, MSV, SMV, MMV) and invisible (0.00 Weber contrast; SSI, MSI, SMI, MMI) expanding disc by running a GLMM with the response to the expanding disc as a binary response variable (0: not responded; 1: responded), disc visibility as a two‐level fixed effect (visible vs. invisible) and individual crab ID as a random effect. As none of the four treatments with invisible discs resulted in statistically different proportions of responding crabs (test 1; Table A2) and the overall response probability in treatments with visible discs was higher than in treatments with invisible discs (test 2; see the Results section), we deemed the response probability to treatments with visible expanding discs to be unaffected by false positive responses and thus removed all treatments with invisible discs (SSI, MSI, SMI and MMI) from further analysis. We tested whether the inclusion or exclusion of the treatments with invisible discs would qualitatively affect the outcome of our subsequent statistical analysis but found that both approaches yielded consistent and statistically similar results.

For the remaining treatments containing visible looming discs (SSV, MSV, SMV and MMV), we tested (3) whether the ability of *C. maenas* to respond is affected solely by the presence of a moving caustic background at the moment of stimulus presentation alone, as well as (4) whether prior exposure to moving caustics affected the likelihood of response even after the caustic movement has stopped. For both test 3 and 4, we ran a GLMM with the response to the expanding disc as a binary response variable (0: not responded; 1: responded), treatment as a fixed effect and individual crab ID as a random effect. To determine (3) whether solely a moving caustic background at the moment of stimulus presentation alone affected the ability of *C. maenas* to respond, we calculated the pairwise contrasts between the SSV and SMV treatments, as well as the MSV and MMV treatments. In both comparisons (SSV vs. SMV; MSV vs. MMV), the two corresponding treatments only differed in whether the caustics were moving or static when the looming stimulus was shown in the stimulus period. This allowed us to test whether exposure to a moving background solely during the presentation of the expanding disc affected the likelihood of responding to that looming stimulus. To establish (4) whether prior exposure to moving caustics alone affected the likelihood of response after the caustic movement has stopped, we calculated the pairwise contrasts between the SSV and MSV treatments, as well as the SMV and MMV treatments. In these comparisons, the two corresponding treatments only varied in the caustic conditions of the exposure period (static or moving). Therefore, any differences in the likelihood of responding to a looming stimulus between these treatments would indicate a historical effect of moving caustics on visual perception.

For Experiment 2, we also established whether changes in caustic condition in the absence of the visible disc elicited responses in the crabs (i.e., false positives). To do so, we tested (5) whether the proportion of responding crabs differed between treatments with a visible (−0.19 Weber contrast; SSV0, MSV0–MSV5) and invisible (0.00 Weber contrast; MSI0) disc by running a GLMM with the response to the expanding disc as a binary response variable (0: not responded; 1: responded), disc visibility as a two‐level fixed effect (visible vs. invisible) and individual crab ID as a random effect. As the response probability in treatments with visible discs was higher than in treatments with invisible discs (see the Results section), we deemed the response probability to treatments with visible expanding discs to be unaffected by false positive responses and thus removed the treatment with an invisible disc (MSI0) from further analysis. We tested whether the inclusion or exclusion of the MSI0 treatment would qualitatively affect the outcome of our subsequent statistical analysis but found that both approaches yielded consistent and statistically similar results. For the remaining treatments containing visible looms (SSV0, MSV0–MSV5), we then established (6) how long a prior exposure to moving caustics affected the response probability in *C. maenas* . To do so, we ran a GLMM with the response to the expanding disc as a binary response variable (0: not responded; 1: responded), treatment as a fixed effect and individual crab ID as a random effect. Subsequently, we calculated the pairwise contrasts between SSV0 (continuous static caustic exposure) and each of the six MSV treatments (MSV0–MSV5) to determine when there was no statistical difference in the proportion of responding crabs between the treatments.

### 2.9 Ethical Statement

The experiment outlined in this study adhered to the ASAB/ASB guidelines for use of animals in behavioural research, was conducted in accordance with UK legislation and was approved by the Animal Welfare and Ethics Review Body of the University of Bristol (UIN/21/061).

## 3 Results

### 3.1 Experiment 1

**FIGURE 2:**
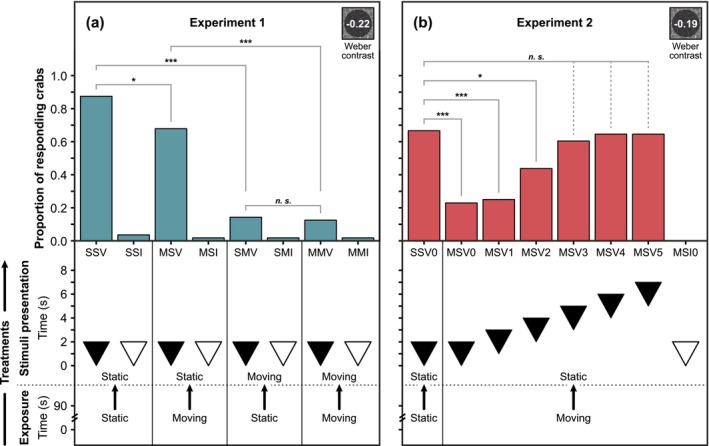
Object response rates in *Carcinus maenas* . (a) Proportion of *C. maenas* (*n* = 56) responding to an approaching predator‐like expanding disc stimulus in the eight treatments of Experiment 1. The false positive response rate of *C. maenas* to the four treatments with invisible discs (0.00 Weber contrast; SSI, MSI, SMI and MMI) was approximately 2% (five responses to 224 presented invisible discs). There was no difference in the proportion of responding crabs between the four treatments with invisible discs (Table A2). Furthermore, the proportion of responding crabs in the treatments with visible discs (−0.22 Weber contrast; SSV, MSV, SMV and MMV) was higher than in the treatments with invisible discs (GLMM: LRT_1_ = 135.91, *p* < 0.001). (b) Proportion of *C. maenas* (*n* = 48) responding to the predator‐like expanding disc stimulus in the eight treatments of Experiment 2. The false positive response rate was 0% (0 responses out of 48 presented invisible discs), and all treatments with a visible disc (SSV0, MSV0–MSV5) resulted in a higher response rate than the MSI0 treatment with no visible disc (GLMM: LRT_1_ = 66.47, *p* < 0.001). (a, b) Treatment name abbreviations: The first and second letters refer to the caustic condition in the exposure and stimulus period (‘M’ for moving caustics; ‘S’ for static caustics), and the third letter states the visibility of the expanding disc (‘V’ for a visible disc with a Weber contrast of −0.22 (Experiment 1) or −0.19 (Experiment 2); ‘I’ for an invisible disc with a Weber contrast of 0.00). In (b) Experiment 2, the additional digit in fourth position refers to the offset (in seconds) at which the disc started to expand (in relation to the start of the stimulus period). Each treatment is visualised underneath the corresponding treatment name. Triangles depict the expanding disc phase, with the lower side of the triangle referring to the time offset of when the disc expansion phase started (in relation to the start of the disc presentation period) and the upper side to the time point when the expanding disc reached its full size. Filled triangles refer to a visible expanding disc stimulus (Experiment 1: −0.22 Weber contrast; Experiment 2: −0.19 Weber contrast), whereas void triangles refer to an invisible expanding disc stimulus (0.00 Weber contrast in both experiments). Significance between treatments is indicated as *** (*p* < 0.001), ** (*p* < 0.01), * (*p* < 0.05) or n.s. (non‐significant).

For the four treatments with visible discs (SSV, MSV, SMV and MMV), exposure to moving caustic backgrounds affected the crabs' likelihood to respond to the predator‐like stimuli (GLMM: LRT_3_ = 108.76; *p* < 0.001; Figure 2a). Fewer crabs responded to the expanding discs when exposed to moving caustics during stimulus presentation, compared to exposure to static caustics during stimulus presentation (SSV vs. SMV: z.ratio = 6.76, *p* < 0.001; MSV vs. MMV: z.ratio = 5.47, *p* < 0.001; Figure 2a; Table A3). When exposed to moving caustics at the time of disc presentation, additional prior exposure to moving caustics in the test period did not affect the likelihood of responding to the stimuli (SMV vs. MMV, z.ratio = 0.28, *p* = 0.781; Figure 2a; Table A3). Therefore, the presence of caustic background motion at the moment of cue presentation alone affects crabs' ability to respond to a looming stimulus, regardless of prior exposure to caustics. In addition, even when there was a static caustic background at the moment of the disc presentation, crabs were less likely to respond to these expanding discs when they had previously experienced moving caustics (SSV vs. MSV; z.ratio = 2.43; *p* = 0.02; Figure 2a; Table A3). Prior exposure to moving caustic background motion, therefore, reduced crabs' likelihood of responding to the looming stimulus even after the caustics had stopped.

### 3.2 Experiment 2

Given that prior exposure to moving caustics reduced the response rate of crabs to expanding discs even after the caustics had ceased (Experiment 1, Figure 2a), in Experiment 2, we tested how long this effect persisted. The temporal offset between the end of the moving caustic exposure and the beginning of the disc presentation affected the likelihood of *C. maenas* to respond to the disc stimuli (GLMM: LRT_6_ = 54.20; *p* < 0.001). Prior exposure to moving caustics impaired the ability of shore crabs to respond to predator‐like expanding discs presented up to 2 s after the caustic background motion had ceased (SSV0 vs. MSV0: z = 4.49, *p* < 0.001; SSV0 vs. MSV1: z = 4.29, *p* < 0.001; SSV0 vs. MSV2: z = 2.43, *p* = 0.03; Figure 2b; Table A4). However, there was no difference in the proportion of crabs responding to expanding discs starting at least 3 s after the end of the moving caustic display, compared to the static control treatment SSV0 (Figure 2b; Table A4). Our findings show that prior exposure to caustics can reduce the likelihood of *C. maenas* responding to a low‐contrast stimulus (−0.19 Weber contrast) mimicking an approaching predator by approximately 65% (32 out of 48 [SSV0] vs. 11 out of 48 responding crabs [MSV0]; Figure 2b) immediately after the caustics have ceased, with this effect lasting at least 2 s for this type of stimulus.

## 4 Discussion

Caustic background motion reduced the response rate of *C. maenas* to an approaching predator‐like stimulus. While our findings confirm recent observations that dynamically moving caustics can impair the ability of aquatic species to respond to moving objects (Attwell et al. 2021; Matchette et al. 2020; Venables et al. 2022), we also established that this reduced visual perception was predominantly caused by the background motion at the moment of object presentation alone, irrespective of whether or not crabs were exposed to moving caustics beforehand. Additionally, we established that prior exposure to moving caustics had a persisting effect on the likelihood of responding to a moving object in *C. maenas* even after the moving caustic exposure had ceased. In particular, crabs showed reduced response rates for approaching stimuli initiated up to 2 s after the termination of the moving caustics. Our study therefore shows that not only the presence of caustic background motion can disrupt visually guided tasks in *C. maenas* but also prior exposure to caustics can reduce the likelihood to respond to visual cues, even after caustic exposure has ceased.

Although caustics can impair the object response rates of aquatic animals (present study; Attwell et al. 2021; Matchette et al. 2020; Venables et al. 2022), the exact properties of caustics that lead to these decreased visual capabilities are still not fully understood. Caustic light bands can affect the visual scene by creating background motion. While background motion might decrease a visual system's ability to segregate spatiotemporal cues of a targeted object against its background (Churan and Ilg 2002), it might also act as a constraint on cognitive processes by distracting or misleading an observer's attention (Dominoni et al. 2020). Many animals have therefore evolved mechanisms that allow them to mitigate the impacts motion might have on their perception (Clifford et al. 2007; Kohn 2007; Webster 2015). For example, visual systems might undergo different types of motion adaptation, defined as a temporary decrease of neural activity to a constant stimulus (Barlow and Hill 1963). By adapting to a constant motion stimulus, visual systems can nullify this stimulus by setting it as the expected norm of the visual scene, thereby increasing the likelihood of detecting other motion stimuli that are different or novel to the adapted visual scene (e.g., an approaching predator against a moving background) (Webster 2015). Considering that caustics lack a directional component at broad spatial and temporal scales, with light bands moving in all directions across the substrate, *C. maenas* might perform a motion gain reduction, similar to the contrast gain reduction observed in flies (Harris et al. 2000), to adapt to the multidirectional background motion provided by caustics. This motion adaptation might aid in preventing saturation of the motion detection neurons, thereby potentially allowing crabs to somewhat reduce the impact of caustic background motion by attempting to increase their sensitivity to other forms of motion. Contrast gain reduction in flies is a rapid visual adaptation and can occur in less than 35 ms after exposure to a moving stimulus (Nordström et al. 2011). If *C. maenas* thus underwent a similar adaptation type in response to caustic background motion, it likely occurred on such a short time frame (tens to hundreds of milliseconds) that is undetectable by our behavioural observations (and would instead require intracellular measurements), explaining why we did not establish a difference in the response rate between the SMV and MMV treatments.

Another dynamic aspect that caustics impose on an animal's visual scene are fluctuations in the intensity of light. Caustic light bands can produce peaks in illumination when moving across the visual background (Drerup, Dunkley, et al. 2024; Venables et al. 2022) or directly across an animal's eye. Crustaceans exposed to caustics might therefore adapt their visual systems by reducing the amount of incoming light, for example, through the migration of screening pigment granules (Meyer‐Rochow 1999, 2001). However, reducing light intake might decrease the visual sensitivity of *C. maenas* (Brodrick et al. 2022). Although the crabs tested in our experimental set‐up were continuously exposed to caustic backgrounds, those backgrounds varied between being either stationary or moving. As stationary caustic backgrounds produce only a few spatially distributed yet non‐moving peaks in illumination and assuming that crabs do not move their eyes in response to static caustic exposure, only the retinal areas that are exposed to these illumination peaks within the visual field would need to perform a brightness adaptation. Contrarily, for moving caustic backgrounds, the peaks in illumination move across the whole visual field, thereby requiring larger areas of the crab's retina to adapt to the increased brightness levels. Moving caustics might therefore reduce the visual sensitivity of a larger proportion of the crab's visual system, potentially explaining the reduced response rate to our predator‐like stimulus against moving caustic backgrounds.

While the visual system of *C. maenas* potentially undergoes adaptations in response to moving caustics, examples from human vision have shown that visual adaptations might also affect visual systems even after the exposure to the adapter has ended (Thompson and Burr 2009) for up to 14 s (Ashida and Osaka 1994). The most notable example of such an after‐effect is the ‘waterfall illusion’ (Addams 1834), which describes that after a prolong exposure to a visual scene moving in a certain direction (e.g., a waterfall), stationary objects can appear to move in the opposite direction (Anstis et al. 1998). Considering that after being exposed to moving caustics, the response probability of *C. maenas* to detect a predator‐like stimuli was reduced for up to 2 s, it is conceivable that this temporary reduction in the ability to respond to an object resulted from an after‐effect caused by a visual adaptation in response to the previous exposure to caustics. Previously described motion after‐effects, such as the waterfall illusion, result from exposure to a visual stimulus moving in one direction (Thompson and Burr 2009). Therefore, caustics are likely unable to elicit the same neural responses due to their multidirectional movement. If *C. maenas* , however, performed a motion gain reduction in response to caustic motion, this visual adaptation might potentially result in a historical visual after‐effect, which could have caused the reduced response rate to our predator‐like stimuli in this species. Moreover, it is also conceivable that a potential brightness adaptation to moving caustics might elicit an after‐effect that resulted in a reduced response rate to our disc stimuli. Future research, therefore, should establish which visual properties of caustic exposure affect animal visual systems both during and after exposure to caustics.

While the neural basis of visual after‐effects and their theoretical benefits and consequences on visual systems have recently been investigated (Glasser et al. 2011; Webster 2015), previous work often focused on standardised yet ecologically unrealistic motion stimuli that an animal would not experience in its natural environment, such as drifting sine wave gratings (e.g., Glasser et al. 2011; Kohn and Movshon 2003). In contrast, our study demonstrated that background motion induced by caustic lighting patterns, a common form of dynamic illumination in shallow marine habitats, might induce visual after‐effects in shore crabs that can reduce their object detection. In particular, our experimental set‐up mimics a scenario in which an animal is approached by a predator against a vertical or sloped background, such as may be found in shallow coastal habitats or large rockpools. Whether this historical effect of exposure to caustics has any consequences for the predation risk of *C. maenas* , or merely represents a by‐product of visual adaptation, remains unknown. In particular, capitalising on the historical effect that caustics might have on prey visual systems might only be feasible for predators that are unaffected by caustics, such as airborne avian predators whose visual perception is likely unimpeded by caustics when searching for prey from above the water surface. These predators could time their foraging bouts to attack prey, such as *C. maenas* , in the brief moments after caustics have ceased to benefit from the reduced object detection rates of their prey. Future research should establish whether predators in the wild can indeed improve their hunting success by targeting their prey in the brief moments after caustics have ended. Alternatively, an adapted version of our experimental setup could be used to expose real predators to both caustic exposure and computer‐generated prey stimuli to test whether these predators time their foraging bouts to the brief periods after caustics have ended.

The findings of the present study open up interesting research avenues to further investigate how caustic exposure and background motion in general affect animal visual systems. For example, crabs in our experimental set‐up were exposed to a fixed duration of > 90 s of caustic background motion, whereas in natural habitats, periods of caustic exposure can vary in length from a few seconds up to several hours. Therefore, future research could explore the effects of both shorter and longer exposure times on the historical effect of caustics, for example, to establish the minimum exposure time needed to elicit this visual adaptation or to determine whether a longer exposure period extends the duration of this historical effect. Another potential research direction could be exploring whether, and to what extent, the intensity level and movement speed of caustics affect the presence and duration of historical effects in animal visual systems, and how those parameters interact with analogous changes in the presented disc stimulus (e.g., difference in contrast strength and expanding speed of the predator‐like stimulus). For example, if a crab's visual system exposed to a moving caustic background adapts to the average perceived motion speed in the visual scene, an observed historical effect in the form of a reduced object response rate might be weaker or last for a shorter duration if the speed of the approaching objects deviates significantly from the adapted speed.

In summary, our findings demonstrate that moving caustics presented only at stimulus presentation can reduce the ability of *C. maenas* to respond to these stimuli; however, prior exposure to caustics in addition to this caustic exposure does neither increase nor decrease the response rate to predator‐like stimuli in this species. Additionally, we found that prior exposure to caustics can have a short‐term historical effect on the visual perception in *C. maenas* , reducing its visual response rates significantly for at least two seconds after the caustics have terminated. Our study motivates future work on how naturally occurring background motion affects the visual systems of animals and which sensory or behavioural adaptations animals have evolved to mitigate or exploit historical effects on visual perception.

## Author Contributions

**Christian Drerup:** conceptualization (equal), data curation (lead), formal analysis (lead), funding acquisition (equal), investigation (lead), methodology (equal), project administration (equal), software (lead), validation (equal), visualization (lead), writing – original draft (lead), writing – review and editing (equal). **James E. Herbert‐Read:** conceptualization (equal), formal analysis (supporting), funding acquisition (equal), methodology (equal), project administration (equal), resources (equal), supervision (equal), validation (equal), writing – review and editing (equal). **Martin J. How:** conceptualization (equal), formal analysis (supporting), funding acquisition (equal), methodology (equal), project administration (equal), resources (lead), software (lead), supervision (equal), validation (equal), writing – review and editing (equal).

## Conflicts of Interest

The authors declare no conflicts of interest.

## Data Availability

All data and code used in this study are deposited on Dryad: https://doi.org/10.5061/dryad.280gb5n17.

## Appendix

**TABLE A1:** Caustic Generator Pro settings used to render the caustic animation.

Menu	Parameter	Setting(s)
Image	Image size	1920 px
	Number of frames	200
	Background colour	127
	Output	BMP
Quality	Resolution	512
	Oversampling	1
	Motion blur samples	1
Pattern	Depth	5 m, 3 m, 1 m
	Intensity	0.05
	Amplitude filter	34.44444
	Frequency filter	4.561728
	Time filter	55
Spectral	Spectral sampling	Off
	Refraction spread	0.08
	Light angle	0°

**TABLE A2:** Pairwise comparison for the treatments with invisible discs (0.00 Weber contrast) in Experiment 1.

Contrast	Estimate	SE	df	z.ratio	*p*
SSI vs. MSI	0.71	1.24	inf.	0.58	1.000
SSI vs. SMI	0.71	1.24	inf.	0.58	1.000
SSI vs. MMI	0.71	1.24	inf.	0.58	1.000
MSI vs. SMI	0.00	1.43	inf.	0.00	1.000
MSI vs. MMI	0.00	1.43	inf.	0.00	1.000
SMI vs. MMI	0.00	1.43	inf.	0.00	1.000

*Note:* Treatment name codes are as described in the Methods section.

**TABLE A3:** Pairwise comparison for the treatments with visible discs (−0.22 Weber contrast) in Experiment 1.

Contrast	Estimate	SE	df	z.ratio	*p*
SSV vs. SMV	3.77	0.56	inf.	6.76	< 0.001
MSV vs. MMV	2.72	0.50	inf.	5.47	< 0.001
SMV vs. MMV	0.16	0.56	inf.	0.28	0.781
SSV vs. MSV	1.21	0.50	inf.	2.43	0.020

*Note:* Treatment name codes are as described in the Methods section.

**TABLE A4:** Pairwise comparison between the SSV0 treatment and all other treatments containing a visible disc (−0.19 Weber contrast) in Experiment 2.

Contrast	estimate	SE	df	z.ratio	*p*
SSV0 vs. MSV0	2.25	0.50	inf.	4.49	< 0.001
SSV0 vs. MSV1	2.11	0.49	inf.	4.29	< 0.001
SSV0 vs. MSV2	1.12	0.46	inf.	2.43	0.030
SSV0 vs. MSV3	0.32	0.46	inf.	0.69	0.735
SSV0 vs. MSV4	0.11	0.47	inf.	0.23	0.816
SSV0 vs. MSV5	0.11	0.47	inf.	0.23	0.816

*Note:* Treatment name codes are as described in the Methods section.

## References

[ece371426-bib-0001] Addams, R. 1834. “An Account of a Peculiar Optical Phænomenon Seen After Having Looked at a Moving Body.” London, Edinburgh, and Dublin Philosophical Magazine and Journal of Science 5: 373–374.

[ece371426-bib-0002] Anstis, S. , F. A. J. Verstraten , and G. Mather . 1998. “The Motion Aftereffect.” Trends in Cognitive Sciences 2: 111–117. 21227087 10.1016/s1364-6613(98)01142-5

[ece371426-bib-0003] Ashida, H. , and N. Osaka . 1994. “Difference of Spatial Frequency Selectivity Between Static and Flicker Motion Aftereffects.” Perception 23: 1313–1320. 7761242 10.1068/p231313

[ece371426-bib-0004] Attwell, J. R. , C. C. Ioannou , C. R. Reid , and J. E. Herbert‐Read . 2021. “Fish Avoid Visually Noisy Environments Where Prey Targeting Is Reduced.” American Naturalist 198: 421–432. 10.1086/715434 34403312

[ece371426-bib-0006] Barlow, H. B. , and R. M. Hill . 1963. “Evidence for a Physiological Explanation of the Waterfall Phenomenon and Figural After‐Effects.” Nature 200: 1345–1347. 14098503 10.1038/2001345a0

[ece371426-bib-0007] Bates, D. , M. Mächler , B. Bolker , and S. Walker . 2015. “Fitting Linear Mixed‐Effects Models Using lme4.” Journal of Statistical Software 67: 1–48.

[ece371426-bib-0008] Benjamini, Y. , and Y. Hochberg . 1995. “Controlling the False Discovery Rate: A Practical and Powerful Approach to Multiple Testing.” Journal of the Royal Statistical Society: Series B: Methodological 57: 289–300.

[ece371426-bib-0009] Brodrick, E. A. , M. J. How , and J. M. Hemmi . 2022. “Fiddler Crab Electroretinograms Reveal Vast Circadian Shifts in Visual Sensitivity and Temporal Summation in Dim Light.” Journal of Experimental Biology 225: jeb243693. 35156128 10.1242/jeb.243693 PMC8976941

[ece371426-bib-0010] Bruno, M. S. , M. I. Mote , and T. H. Goldsmith . 1973. “Spectral Absorption and Sensitivity Measurements in Single Ommatidia of the Green Crab, *Carcinus* .” Journal of Comparative Physiology 82: 151–163.

[ece371426-bib-0011] Calanni, J. S. , M. L. Aranda , H. H. Dieguez , D. Dorfman , T. M. Schmidt , and R. E. Rosenstein . 2024. “An Ethologically Relevant Paradigm to Assess Defensive Response to Looming Visual Contrast Stimuli.” Scientific Reports 14: 12499. 38822033 10.1038/s41598-024-63458-1 PMC11143276

[ece371426-bib-0012] Churan, J. , and U. J. Ilg . 2002. “Flicker in the Visual Background Impairs the Ability to Process a Moving Visual Stimulus.” European Journal of Neuroscience 16: 1151–1162. 12383245 10.1046/j.1460-9568.2002.02164.x

[ece371426-bib-0013] Clifford, C. W. G. , M. A. Webster , G. B. Stanley , et al. 2007. “Visual Adaptation: Neural, Psychological and Computational Aspects.” Vision Research 47: 3125–3131. 17936871 10.1016/j.visres.2007.08.023

[ece371426-bib-0014] Cronin, T. W. , and K. D. Feller . 2014. “Sensory Ecology of Vision in Crustaceans.” In Crustacean Nervous Systems and Their Control of Behavior, edited by C. Derby and M. Thiel , 235–262. Oxford University Press.

[ece371426-bib-0015] Crothers, J. 1968. “The Biology of the Shore Crab, *Carcinus maenas* (L.). 2. The Life of the Adult Crab.” Field Studies 2: 579–614.

[ece371426-bib-0016] Dominoni, D. M. , W. Halfwerk , E. Baird , et al. 2020. “Why Conservation Biology Can Benefit From Sensory Ecology.” Nature Ecology & Evolution 4: 502–511. 32203474 10.1038/s41559-020-1135-4

[ece371426-bib-0017] Drerup, C. , K. Dunkley , M. J. How , and J. E. Herbert‐Read . 2024. “Cuttlefish Adopt Disruptive Camouflage Under Dynamic Lighting.” Current Biology 34: 3258–3264. 38959882 10.1016/j.cub.2024.06.015

[ece371426-bib-0018] Drerup, C. , and M. J. How . 2021. “Polarization Contrasts and Their Effect on the Gaze Stabilization of Crustaceans.” Journal of Experimental Biology 224: jeb229898. 10.1242/jeb.229898 PMC8077661 33692078

[ece371426-bib-0019] Drerup, C. , M. J. How , and J. E. Herbert‐Read . 2023. “Visual Noise From Caustic Flicker Does Not Affect the Hunting Success of Cuttlefish.” Animal Behaviour 202: 59–72.

[ece371426-bib-0020] Drerup, C. , M. J. How , and J. E. Herbert‐Read . 2024. “Dynamic Visual Noise has Limited Influence on the Habitat Selection and Behavioural Activity of Crustaceans and Cephalopods.” Ethology 130: e13432.

[ece371426-bib-0021] Eckert, M. P. , and J. Zeil . 2001. “Towards an Ecology of Motion Vision.” In Motion Vision: Computational, Neural, and Ecological Constraints, edited by J. M. Zanker and J. Zeil , 333–369. Springer Berlin Heidelberg.

[ece371426-bib-0022] Gallagher, R. M. , T. Suddendorf , and D. H. Arnold . 2021. “The Implied Motion Aftereffect Changes Decisions, but Not Confidence.” Attention, Perception, & Psychophysics 83: 3047–3055. 10.3758/s13414-021-02331-z PMC8550352 34427903

[ece371426-bib-0023] Glasser, D. M. , J. M. G. Tsui , C. C. Pack , and D. Tadin . 2011. “Perceptual and Neural Consequences of Rapid Motion Adaptation.” Proceedings of the National Academy of Sciences 108: E1080–E1088. 10.1073/pnas.1101141108 PMC3215073 21709221

[ece371426-bib-0024] Grober, M. S. 1990. “Luminescent Flash Avoidance in the Nocturnal Crab *Portunus Xantusii*: II. Cardiac and Visual Responses to Variations in Simulated Luminescent Flashes.” Journal of Experimental Biology 148: 427–448.

[ece371426-bib-0025] Harris, R. A. , D. C. O'Carroll , and S. B. Laughlin . 2000. “Contrast Gain Reduction in Fly Motion Adaptation.” Neuron 28: 595–606. 11144367 10.1016/s0896-6273(00)00136-7

[ece371426-bib-0026] Hartig, F. 2022. “DHARMa: Residual Diagnostics for Hierarchical (Multi‐Level/Mixed) Regression Models.” R Package Version 0.4.5. https://cran.r‐project.org/package=DHARMa.

[ece371426-bib-0027] Horseman, B. G. , M. W. S. Macauley , and W. J. P. Barnes . 2011. “Neuronal Processing of Translational Optic Flow in the Visual System of the Shore Crab *Carcinus maenas* .” Journal of Experimental Biology 214: 1586–1598. 21490266 10.1242/jeb.050955

[ece371426-bib-0028] Kohn, A. 2007. “Visual Adaptation: Physiology, Mechanisms, and Functional Benefits.” Journal of Neurophysiology 97: 3155–3164. 17344377 10.1152/jn.00086.2007

[ece371426-bib-0029] Kohn, A. , and J. A. Movshon . 2003. “Neuronal Adaptation to Visual Motion in Area MT of the Macaque.” Neuron 39: 681–691. 12925281 10.1016/s0896-6273(03)00438-0

[ece371426-bib-0030] Land, M. F. , and D.‐E. Nilsson . 2012. Animal Eyes. Oxford University Press.

[ece371426-bib-0031] Layne, J. E. , M. Wicklein , F. A. Dodge , and R. B. Barlow . 1997. “Prediction of Maximum Allowable Retinal Slip Speed in the Fiddler Crab, *Uca pugilator* .” Biological Bulletin 193: 202–203. 9390385 10.1086/BBLv193n2p202

[ece371426-bib-0032] Lenth, R. 2022. “emmeans: Estimated Marginal Means, Aka Least‐Squares Means.” R Package Version 1.9.0. https://cran.r‐project.org/package=emmeans.

[ece371426-bib-0033] Lock, J. A. , and J. H. Andrews . 1992. “Optical Caustics in Natural Phenomena.” American Journal of Physics 60: 397–407.

[ece371426-bib-0034] Matchette, S. R. , I. Cuthill , K. Cheney , N. Marshall , and N. Scott‐Samuel . 2020. “Underwater Caustics Disrupt Prey Detection by a Reef Fish.” Proceedings of the Royal Society B 287: 20192453. 32228405 10.1098/rspb.2019.2453 PMC7209061

[ece371426-bib-0035] Matchette, S. R. , I. C. Cuthill , and N. E. Scott‐Samuel . 2018. “Concealment in a Dynamic World: Dappled Light and Caustics Mask Movement.” Animal Behaviour 143: 51–57.

[ece371426-bib-0036] Matchette, S. R. , I. C. Cuthill , and N. E. Scott‐Samuel . 2019. “Dappled Light Disrupts Prey Detection by Masking Movement.” Animal Behaviour 155: 89–95.

[ece371426-bib-0037] Meyer‐Rochow, V. B. 1999. “Compound Eye: Circadian Rhythmicity, Illumination, and Obscurity.” In Atlas of Arthropod Sensory Receptors, edited by E. Eguchi and Y. Tominaga , 97–125. Springer.

[ece371426-bib-0038] Meyer‐Rochow, V. B. 2001. “The Crustacean Eye: Dark/Light Adaptation, Polarization Sensitivity, Flicker Fusion Frequency, and Photoreceptor Damage.” Zoological Science 18: 1175–1197. 11911074 10.2108/zsj.18.1175

[ece371426-bib-0039] Nordström, K. , I. M. de Miguel , and D. C. O'Carroll . 2011. “Rapid Contrast Gain Reduction Following Motion Adaptation.” Journal of Experimental Biology 214: 4000–4009. 22071192 10.1242/jeb.057539

[ece371426-bib-0041] R Core Team . 2023. R: A Language and Environment for Statistical Computing. R Foundation for Statistical Computing. https://www.r‐project.org/.

[ece371426-bib-0042] Schiff, W. , J. A. Caviness , and J. J. Gibson . 1962. “Persistent Fear Responses in Rhesus Monkeys to the Optical Stimulus of “Looming”.” Science 136: 982–983. 14498362 10.1126/science.136.3520.982

[ece371426-bib-0043] Srinivasan, M. V. , M. Poteser , and K. Kral . 1999. “Motion Detection in Insect Orientation and Navigation.” Vision Research 39: 2749–2766. 10492835 10.1016/s0042-6989(99)00002-4

[ece371426-bib-0044] Thompson, P. , and D. Burr . 2009. “Visual Aftereffects.” Current Biology 19: R11–R14. 19138580 10.1016/j.cub.2008.10.014

[ece371426-bib-0045] Venables, S. V. , C. Drerup , S. B. Powell , N. J. Marshall , J. E. Herbert‐Read , and M. J. How . 2022. “Polarization Vision Mitigates Visual Noise From Flickering Light Underwater.” Science Advances 8: eabq2770. 36083913 10.1126/sciadv.abq2770 PMC9462692

[ece371426-bib-0046] Wald, G. 1968. “Single and Multiple Visual Systems in Arthropods.” Journal of General Physiology 51: 125–156. 5641632 10.1085/jgp.51.2.125 PMC2201124

[ece371426-bib-0047] Webster, M. A. 2015. “Visual Adaptation.” Annual Review of Vision Science 1: 547–567. 10.1146/annurev-vision-082114-035509 PMC4742349 26858985

[ece371426-bib-0048] Wickham, H. 2016. ggplot2: Elegant Graphics for Data Analysis. Springer.

[ece371426-bib-0049] Zeil, J. , G. Nalbach , and H. O. Nalbach . 1986. “Eyes, Eye Stalks and the Visual World of Semi‐Terrestrial Crabs.” Journal of Comparative Physiology A 159: 801–811.

